# Burnout Among Mid-Career Academic Medical Faculty

**DOI:** 10.1001/jamanetworkopen.2024.15593

**Published:** 2024-06-10

**Authors:** Kelly C. Paradis, Eve A. Kerr, Kent A. Griffith, Christina M. Cutter, Eva L. Feldman, Kanakadurga Singer, Nancy D. Spector, Peter A. Ubel, Reshma Jagsi

**Affiliations:** 1 Department of Radiation Oncology, University of Michigan, Ann Arbor; 2 Department of Internal Medicine, University of Michigan, Ann Arbor; 3 VA Center for Clinical Management Research, Department of Veterans Affairs, Ann Arbor, Michigan; 4 Center for Cancer Data Sciences, University of Michigan School of Public Health, Ann Arbor; 5 Center for Bioethics and Social Sciences in Medicine, University of Michigan, Ann Arbor; 6 Department of Emergency Medicine, University of Michigan, Ann Arbor; 7 Department of Neurology, University of Michigan, Ann Arbor; 8 Department of Pediatrics, University of Michigan, Ann Arbor; 9 Department of Pediatrics and Lynn Yeakel Institute for Women’s Health and Leadership, Drexel University College of Medicine, Philadelphia, Pennsylvania; 10 Schools of Business, Public Policy and Medicine, Duke University, Durham, North Carolina; 11 Department of Radiation Oncology, Emory University School of Medicine and Winship Cancer Institute, Atlanta, Georgia

## Abstract

**Importance:**

Studies reveal that most physicians report symptoms of burnout. Less is known about burnout in mid-career medical faculty specifically.

**Objective:**

To characterize burnout and its risk factors, particularly differences by gender, among mid-career medical faculty.

**Design, Setting, and Participants:**

Between August 2021 and August 2022, a survey was sent to 1430 individuals who received new National Institutes of Health K08 and K23 career development awards from 2006 to 2009. Data were analyzed between June and October 2023.

**Main Outcomes and Measures:**

Personal and work-related burnout as evaluated using the Copenhagen Burnout Inventory (CBI). The CBI score ranges from 0 to 100, with a score of 50 or higher indicating a high degree of burnout. Multivariable models were used to investigate associations between burnout and participant characteristics, including race and ethnicity, sexual orientation and gender identity, academic rank, work climate, experiences of workplace sexual harassment, sleep hours, work and domestic caregiving time, and time allocation changes in work and domestic work hours compared with before the COVID-19 pandemic. Work climate was evaluated by a general climate elements scale assessing elements such as friendliness, respect, and collegiality, and a diversity, equity, and inclusion climate elements scale assessing elements such as homogeneity, sexism, and homophobia; higher scores indicated a more favorable view of the climate.

**Results:**

In all, 1430 surveys were sent, 926 candidates responded (65% response rate), and the analytic cohort was limited to the 841 respondents who were still in academic medicine (50.7% men). Burnout was significantly more common for women than men (mean [SD] CBI personal scores, 46.6 [19.4] vs 37.5 [17.2]; *P* < .001; mean [SD] CBI work-related scores, 43.7 [20.4] vs 34.6 [19.7]; *P* < .001). In multivariable models, personal burnout was significantly more likely for women (adjusted odds ratio [AOR], 2.29 [95% CI, 1.54-3.41]; *P* < .001) and with more weekly hours of patient care (AOR, 1.07 [95% CI, 1.00-1.15] for each 5-hour increase; *P* = .04). Personal burnout was less likely with more nightly sleep hours (AOR, 0.68 [95% CI, 0.56-0.81] for each 1-hour increase; *P* < .001) and with an improved general work climate rating (AOR, 0.64 [95% CI, 0.48-0.85] for each 1-point increase in general work climate scale score; *P* = .002). Work-related burnout was also significantly more likely for women than men (AOR, 1.77 [95% CI, 1.17-2.69]; *P* = .007). Greater work-related burnout was associated with an increase of 8 or more work hours per week compared with before the COVID-19 pandemic (AOR, 1.87 [95% CI, 1.13-3.08]; *P* = .01), more weekly hours of patient care (AOR, 1.11 [95% CI, 1.03-1.19] for each 5-hour increase; *P* = .007), and a workplace sexual harassment experience in the past 2 years (AOR, 1.71 [95% CI, 1.11-2.62]; *P* = .01). Work-related burnout was significantly less likely with more nightly sleep hours (AOR, 0.80 [95% CI, 0.66-0.96] for each 1-hour increase; *P* = .02) and with an improved general work climate rating (AOR, 0.49; [95% CI, 0.36-0.65] for each 1-point increase in general work climate scale score; *P* < .001).

**Conclusions and Relevance:**

This survey study of K grant awardees revealed substantial rates of burnout among mid-career medical faculty, and burnout rates differed by gender. Evidence-based interventions are needed to realize the benefits of workforce diversity and vitality.

## Key Points

**Question:**

What is the prevalence of burnout for mid-career medical faculty, and what factors are associated with it?

**Findings:**

In this survey study of 841 individuals who received new National Institutes of Health K08 and K23 awards, work-related burnout was common (23.0% of men and 40.8% of women), and rates remained higher for women after controlling for covariates. Burnout was less likely with an improved general work climate scale rating.

**Meaning:**

Given the substantial rates of burnout and gender differences, evidence-based interventions are necessary to realize the benefits of workforce diversity and vitality.

## Teaser

This survey study examines the prevalence of burnout among mid-career medical faculty and factors associated with personal and work-related burnout.

## Introduction

Concerns about burnout, career dissatisfaction, and work attrition among physicians have been growing, with extensive studies over the past 2 decades reporting that over half of physicians report symptoms of burnout.^1^ Burnout is defined by the National Academy of Sciences, Engineering, and Medicine as “a workplace syndrome characterized by high emotional exhaustion, high depersonalization (eg, cynicism), and a low sense of personal accomplishment.”^1^ Faculty who are women or of racial or ethnic groups underrepresented in medicine (URIM) have higher rates of burnout and attrition compared with faculty outside of these identity groups, but understanding of the factors that might drive these differences is still developing.^1,2^ Burnout among physicians was an issue even before the COVID-19 outbreak, but the pandemic intensified conditions that might lead to burnout,^3,4^ necessitating strategies to maintain the US health care workforce’s capacity and well-being.

Recognizing the implications of these alarming trends, the National Academy of Sciences, Engineering, and Medicine released the National Plan for Health Workforce Well-Being in 2022.^5^ Measuring, understanding, and increasing the visibility of burnout using validated measures were highlighted as priority areas, including understanding the implications of discrimination, sexism, and the COVID-19 pandemic for the US health care workforce.

Research thus far has primarily focused on understanding burnout among clinicians, with less attention to the unique experiences of academic physician-researchers, particularly those in mid-career, who finished their training more than a decade ago. Mid-career may be a particularly vulnerable time for burnout for several reasons, including caregiving responsibilities, decreased mentorship support, and increased service and academic productivity expectations. These challenges may be more acute for women faculty, who often bear the brunt of caregiving responsibilities and experience a phenomenon of invisibility characterized by marginalization and reduced recognition.^6,7^ Conversely, mid-career is also sometimes a time of increased stability by attaining promotion to more senior ranks, national reputation, and broader mentorship networks. Few studies have focused on understanding burnout experiences at this critical stage in academic medical careers or whether burnout is experienced differently by men and women in this stage.

The current study focused on a cohort of mid-career faculty who received prestigious National Institutes of Health K08 or K23 clinician-researcher awards between 2006 and 2009 because considerable societal resources were invested in these high-performing faculty. We have previously studied burnout in this cohort during an earlier phase of their careers^8^; understanding differential levels of burnout by gender over time in this cohort is particularly important as these differences could lead to longer-term disparities in future contributions of these faculty. Therefore, we explored differences in burnout by gender in this cohort now at the mid-career stage. We then tested whether gender differences could be explained by covariates, such as race or ethnicity, sexual orientation and gender identity, work hours, domestic care hours, and work climate, including personal experiences with workplace sexual harassment. We also assessed burnout from work-hour changes after the outbreak of the COVID-19 pandemic, which took a toll on the US health care workforce.

## Methods

### Survey Administration

This study was approved by the University of Michigan Institutional Review Board, which waived participants’ written documentation of consent because the research presents no more than minimal risk of harm and involves no procedures for which written consent is normally required outside of the research context. The survey methods have been described previously^9^ and reflect consideration of the American Association for Public Opinion Research (AAPOR) best practices for questionnaire design, administration, and analysis, including quality assurance and efforts to maximize response rates. Briefly, the survey cohort included clinician-researchers who received new K08 and K23 career development awards between 2006 and 2009. A total of 1430 K grant awardees with verifiable contact information were mailed or emailed invitations to participate in the survey, along with a $50 nonconditional incentive, between August 2021 and August 2022. The analytic cohort was limited to those remaining in academic medicine.

Study data were collected and managed using REDCap tools (Vanderbilt University) hosted at the University of Michigan.^10,11^ REDCap is a secure, web-based software platform designed to support data capture for research studies, providing an intuitive interface for validated data capture, audit trails for tracking data manipulation and export procedures, automated export procedures for seamless data downloads to common statistical packages, and procedures for data integration and interoperability with external sources.

### Survey Development and Key Outcome Measures

The 12-page survey evaluated quality of life, satisfaction with career, academic rank, leadership activities, time allocation, career changes, publications and funding, mentoring, home and family, online experiences, work environment and climate, and well-being. The current report focuses on personal and work-related burnout, as evaluated using the Copenhagen Burnout Inventory (CBI).^12^

The CBI is a frequently used measure to quantify burnout in health care and is a measure recommended by the National Academies of Sciences, Engineering, and Medicine.^1,12,13,14,15^ Personal burnout is evaluated by 6 questions, and work-related burnout by 7 questions, answered on a 5-point Likert scale. The CBI focuses on fatigue and exhaustion as the primary burnout symptoms and attempts to attribute them to specific domains of life. The personal CBI burnout measure quantifies fatigue and exhaustion related to any factor (ie, the word *personal* should not be interpreted as excluding work-related factors), whereas the work-related CBI burnout measure specifically assesses work-related factors. Answers to each question are scored in 25-point increments ranging from 0 to 100 (a higher score indicates a greater degree of burnout); the composite personal or work-related score is an average of the component questions of the measure with 1 item reverse scored because it is written with reversed logic compared with the other items (ie, a stronger agreement indicates less burnout). As in prior studies that used the CBI,^8,16,17,18^ a dichotomized variable was created for both personal and work-related burnout, using a cutoff score of 50 or higher to indicate the presence of burnout.

### Key Independent Variables

Participants were asked to report demographic details and membership in 3 identity groups (multiple options could be selected): gender (woman, man, nonbinary, or decline to answer), sexual orientation (heterosexual/straight, gay/lesbian, bisexual, none [write-in text], or decline to answer), and race and ethnicity (Asian, URIM [including American Indian or Alaska Native, Black or African American, Native Hawaiian or Other Pacific Islander, or multiple races other than Asian and White, which was categorized as Asian], White, or unknown). We used the initialism LGBTQ+ (lesbian, gay, bisexual, transgender, or queer, with the plus holding space for the expanding definition of this term) to represent respondents who do not identify as both cisgender and heterosexual. Data on race and ethnicity were collected to permit evaluation of differences in subjects' experiences that might be associated with identity characteristics. Missing answers were excluded. Several other measures were also assessed that were hypothesized could be associated with burnout, including marital status, spousal employment status, dependent care responsibilities, academic rank, weekly work hours, weekly patient care hours, weekly domestic work hours, nightly sleep hours, satisfaction of mentoring received, general and work climate scales,^19^ and experiences with workplace sexual harassment, along with measures of substantial disruptions in time allocation (increase of ≥8 hr/wk in work hours or ≥8 hr/wk in domestic labor hours) compared with before the outbreak of COVID-19. Work climate was evaluated by a general climate elements scale assessing elements such as friendliness, respect, and collegiality, and a diversity, equity, and inclusion climate elements scale assessing elements such as homogeneity, sexism, and homophobia; higher scores indicated a more favorable view of the climate.

### Statistical Analysis

Descriptive statistics were used to compare demographic characteristics and experiences by gender. For the 2 CBI burnout measures—personal and work-related—differences by gender were first evaluated, as were bivariate associations with selected independent variables. Standard *P* values and adjusted *P* values for multiple testing using the Holm method are reported, with *P* < .05 considered statistically significant.^20^ Fully adjusted multiple variable models were then created for each of the 2 dichotomous burnout measures using the covariates. Adjusted associations estimated from complete case analyses were reported, which entails using only those cases with complete data for the outcome and covariates. To ensure association estimates were not biased due to missing data, sensitivity analyses were conducted (eAppendix in Supplement 1). These analyses compared complete case estimated associations with those estimated after multiple imputation of any missing covariate data using fully conditional specified chain equations (an accepted imputation method when the missing data pattern is unknown) using Stata, version 16.1 (StataCorp, LLC) and SAS, version 9.4 (SAS Institute Inc).^21^ Comparisons were made between both a dichotomous indicator and the continuous burnout scores with both sets of sensitivity analyses (eAppendix in Supplement 1). Data were analyzed from June to October 2023.

## Results

Of 1430 surveys sent, 926 completed surveys were returned (response rate, 65%). Of the 926 respondents, 841 (90.8%) reported that they were still in academia and constituted the study sample (426 men [50.7%], 392 women [46.6%], 2 nonbinary gender [0.2%], and 21 who did not identify gender [2.5%]). Of these 841 individuals, there were 172 Asian respondents (20.5%), 66 URIM respondents (7.8%), 580 White respondents (69.0%), and 23 respondents who did not report race and ethnicity (2.7%). There were 783 respondents (93.1%) who identified as cisgender and heterosexual, 34 had LGBTQ+ status (4.0%), and 24 did not identify sexual orientation (2.9%). The Table provides characteristics and experiences of the analytic cohort by gender.

**Table.** Characteristics and Experiences of Respondents by Gender

Characteristic	Gender category, No. (%)			*P* value, women vs men	Adjusted *P* value, women vs men^a^
	Women (n = 392)	Men (n = 426)	Nonbinary gender or did not indicate (n = 23)		
Gender					
Man	0	426 (100)	0	NA	NA
Woman	392 (100)	0	0	NA	NA
Nonbinary	0	0	2 (8.70)	NA	NA
Missing or not reported	0	0	21 (91.30)	NA	NA
Race and ethnicity					
Asian	83 (21.17)	88 (20.66)	1 (4.35)	.77	.77
Underrepresented in medicine	29 (7.39)	37 (8.69)	0		
White	280 (71.43)	296 (69.48)	4 (17.39)		
Missing or not reported	0	5 (1.17)	18 (78.26)		
Sexual orientation					
Heterosexual or straight	374 (95.41)	409 (96.01)	2 (8.70)	.28	>.99
Gay or lesbian	8 (2.04)	13 (3.05)	0		
Bisexual	6 (1.53)	1 (0.23)	0		
None^b^	1 (0.26)	1 (0.23)	2 (8.70)		
Missing or not reported	3 (0.77)	2 (0.47)	19 (82.61)		
Marital status and spousal employment					
Single, divorced, or widowed	48 (12.24)	22 (5.16)	4 (17.39)	<.001	<.001
Married and spouse or domestic partner not employed or part-time employed	74 (18.88)	186 (43.66)	7 (30.43)		
Married and spouse or domestic partner full-time employed	266 (67.86)	214 (50.23)	10 (43.48)		
Missing or not reported	4 (1.02)	4 (0.94)	2 (8.70)		
Children requiring adult supervision					
No	175 (44.64)	225 (52.82)	15 (65.22)	.02	.14
Yes	213 (54.34)	196 (46.01)	6 (26.09)		
Missing or not reported	4 (1.02)	5 (1.17)	2 (8.70)		
Other dependent(s)					
No	299 (76.28)	352 (82.63)	18 (78.26)	.006	.06
Yes	84 (21.43)	59 (13.85)	2 (8.70)		
Missing or not reported	9 (2.30)	15 (3.52)	3 (13.04)		
Academic rank					
Professor	202 (51.53)	254 (59.62)	10 (43.48)	.04	.30
Associate professor	175 (44.64)	153 (35.92)	0		
Assistant professor or other^c^	15 (3.83)	19 (4.46)	1 (4.35)		
Specialty					
Basic sciences or nonclinical	114 (29.08)	55 (12.91)	5 (21.74)	<.001	<.001
Clinical specialties for women, children, and families	101 (25.77)	88 (20.66)	4 (17.39)		
Hospital-based specialties	34 (8.67)	60 (14.08)	5 (21.74)		
Medical specialties	137 (34.95)	184 (43.19)	9 (39.13)		
Surgical specialties	6 (1.53)	39 (9.15)	0		
Region					
West	102 (26.02)	102 (23.94)	9 (39.13)	.58	>.99
Midwest	77 (19.64)	99 (23.24)	3 (13.04)		
Northeast	128 (32.65)	124 (29.11)	7 (30.43)		
South	83 (21.17)	98 (23.00)	4 (17.39)		
International	2 (0.51)	3 (0.70)	0		
Substantial (>8 hr) disruption in hours spent on work after COVID-19 pandemic					
No	315 (80.36)	356 (83.57)	18 (78.26)	.30	.89
Yes	74 (18.88)	69 (16.20)	4 (17.39)		
Missing or not reported	3 (0.77)	1 (0.23)	1 (4.35)		
Substantial (>8 hr) disruption in hours spent on caregiving or domestic labor after COVID-19 pandemic					
No	303 (77.30)	378 (88.73)	16 (69.57)	<.001	<.001
Yes	83 (21.17)	40 (9.39)	5 (21.74)		
Missing or not reported	6 (1.53)	8 (1.88)	2 (8.7)		
Work hours per week					
No.	389	425	22	.006	.06
Mean (SD) [95% CI]	55.52 (13.36) [54.19-56.85]	58.04 (12.68) [56.84-59.25]	62.27 (15.10) [55.96-68.58]		
Hours spent on caregiving or domestic labor per week					
No.	387	418	21	<.001	<.001
Mean (SD) [95% CI]	30.31 (21.05) [28.21-32.41]	22.99 (16.88) [21.37-24.61]	27.81 (18.83) [19.76-35.86]		
Hours spent on patient care per week					
No.	382	421	22	<.001	<.001
Mean (SD) [95% CI]	13.1 (12.96) [11.80-14.40]	17.67 (14.51) [16.28-19.05]	16.6 (14.37) [10.59-22.60]		
Hours of sleep per night					
No.	388	422	21	.07	.40
Mean (SD) [95% CI]	6.85 (1.11) [6.74-6.97]	6.72 (0.93) [6.63-6.81]	6.36 (1.34) [5.78-6.93]		
Satisfaction with mentoring received					
No	111 (28.32)	100 (23.47)	11 (47.83)	.22	>.99
Yes	268 (68.37)	294 (69.01)	9 (39.13)		
Missing or not reported	13 (3.32)	32 (7.51)	3 (13.04)		
Work climate score, general^d^					
No.	379	423	19	<.001	<.001
Mean (SD) [95% CI]	3.68 (0.92) [3.59-3.77]	3.97 (0.85) [3.89-4.05]	3.20 (0.97) [2.77-3.64]		
Work climate score, DEI^d^					
No.	377	423	19	<.001	<.001
Mean (SD) [95% CI]	3.72 (0.82) [3.63-3.80]	4.17 (0.67) [4.10-4.23]	3.81 (0.90) [3.41-4.21]		
Sexual harassment experienced					
No	107 (27.30)	231 (54.23)	5 (21.74)	<.001	<.001
Yes	281 (71.68)	194 (45.54)	13 (56.52)		
Missing or not reported	4 (1.02)	1 (0.23)	5 (21.74)		

Abbreviations: DEI, diversity, equity, and inclusion; NA, not applicable.

^a^ *P* values adjusted using the Holm method to account for multiple testing.

^b^ Write-in responses were queer (n = 2), man (n = 1), and asexual (n = 1).

^c^ Other includes department chairperson.

^d^ Work climate was evaluated by a general climate elements scale assessing elements such as friendliness, respect, and collegiality, and a diversity, equity, and inclusion climate elements scale assessing elements such as homogeneity, sexism, and homophobia. Higher scores indicate a more favorable view of the climate.

**Figure 1.**
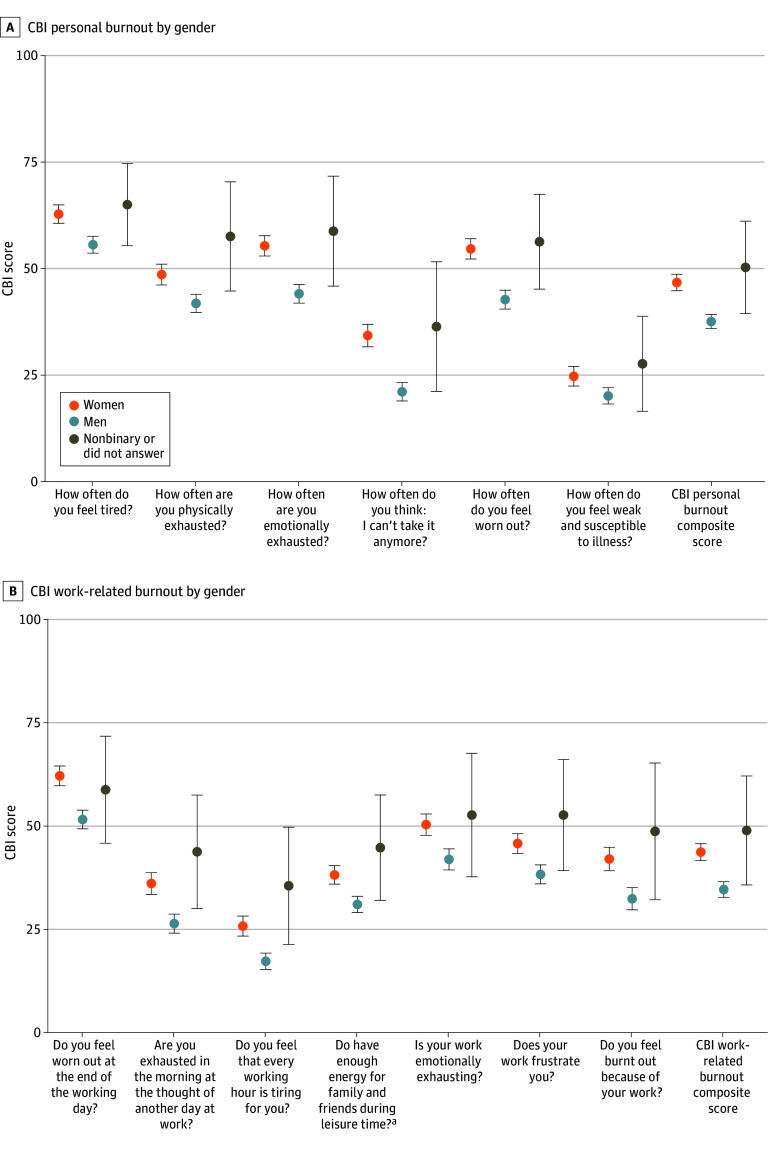
Bivariate Results for Copenhagen Burnout Inventory (CBI) Scores by Gender Circles represent the mean and whiskers indicate 95% CIs. ^a^Item is reverse scored.

Compared with men, women had significantly higher scores on the CBI personal and work-related scales in bivariate analyses. Figure 1A shows CBI personal burnout scores by gender for each item on the scale, as well as the composite CBI personal burnout score; Figure 1B shows the corollary for CBI work-related burnout. On a continuous scale, with higher scores indicating a higher degree of burnout, women and men had composite mean (SD) scores of 46.6 (19.4) vs 37.5 (17.2) (*P* < .001), respectively, for personal burnout and 43.7 (20.4) vs 34.6 (19.7) (*P* < .001), respectively, for work-related burnout. When a cutoff value of 50 was used for these scales, significantly more women than men experienced high levels of both personal burnout (46.7% [183 of 392] vs 25.6% [109 of 426]; *P* < .001) and work-related burnout (40.8% [160 of 392] vs 23.0% [98 of 426]; *P* < .001), respectively.

Bivariate analyses also revealed several other independent variables we considered might be associated with the personal and work-related burnout measures (eAppendix in Supplement 1). For personal burnout, factors associated with an increased risk of burnout included an increase in work of 8 or more hours per week compared with before the outbreak of COVID-19; an increase in domestic labor of 8 or more hours per week compared with before the outbreak of COVID-19; higher weekly work hours, or caregiving/domestic tasks hours; lower nightly sleep hours; dissatisfaction with mentoring received; having a poorer perception of general work climate or work climate related to diversity, equity, and inclusion; and having an experience with workplace sexual harassment in the past 2 years. For example, for those with an experience of workplace sexual harassment, the odds ratio of personal burnout was 2.20 (95% CI, 1.59-3.04) compared with those without this experience.

For work-related burnout, factors associated with an increased risk of burnout were similar to those for personal burnout, but also included higher weekly hours of patient care. For example, for those with an experience of workplace sexual harassment, the odds ratio of work-related burnout was 2.84 (95% CI, 1.99-4.05) compared with those without this experience. Additional details are in the eAppendix in Supplement 1.

**Figure 2.**
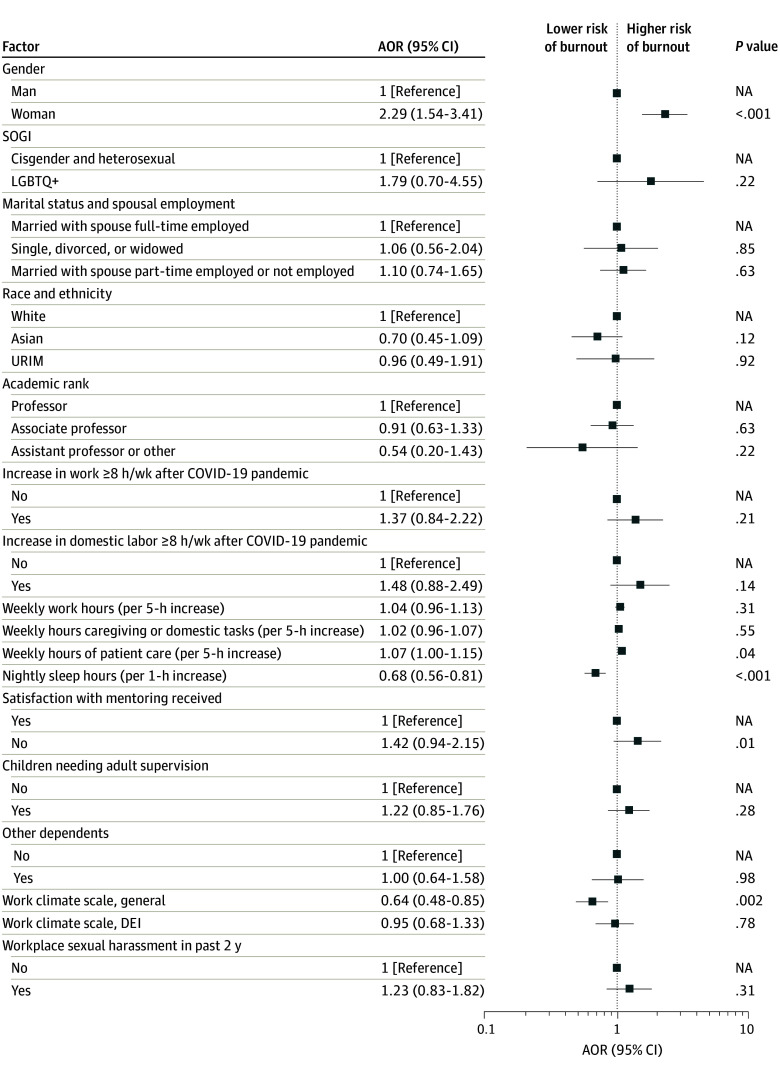
Multivariable Associations of Personal Burnout on the Copenhagen Burnout Inventory Races and ethnicities included in the underrepresented in medicine (URIM) category included American Indian or Alaska Native, Black or African American, Native Hawaiian or Other Pacific Islander, or multiple races other than Asian and White, which was categorized as Asian. AOR indicates adjusted odds ratio; DEI, diversity, equity, and inclusion; LGBTQ+, lesbian, gay, bisexual, transgender, or queer, with the plus holding space for the expanding definition of this term; NA, not applicable; and SOGI, sexual orientation and gender identity.

In a multivariable model of personal burnout (Figure 2), burnout remained significantly more likely for women than men (adjusted odds ratio [AOR], 2.29 [95% CI, 1.54-3.41]; *P* < .001) even after controlling for other covariates hypothesized to potentially be associated with the outcome. Personal burnout was also independently more likely with more weekly hours of patient care (AOR, 1.07 [95% CI, 1.00-1.15] for each 5-hour increase; *P* = .04) and less likely with more nightly sleep hours (AOR, 0.68 [95% CI, 0.56-0.81] for each 1-hour increase; *P* < .001) as well as with higher ratings (ie, more favorable perceptions) for the general work climate (AOR, 0.64 [95% CI, 0.48-0.85] for each 1-point increase; *P* = .002).

**Figure 3.**
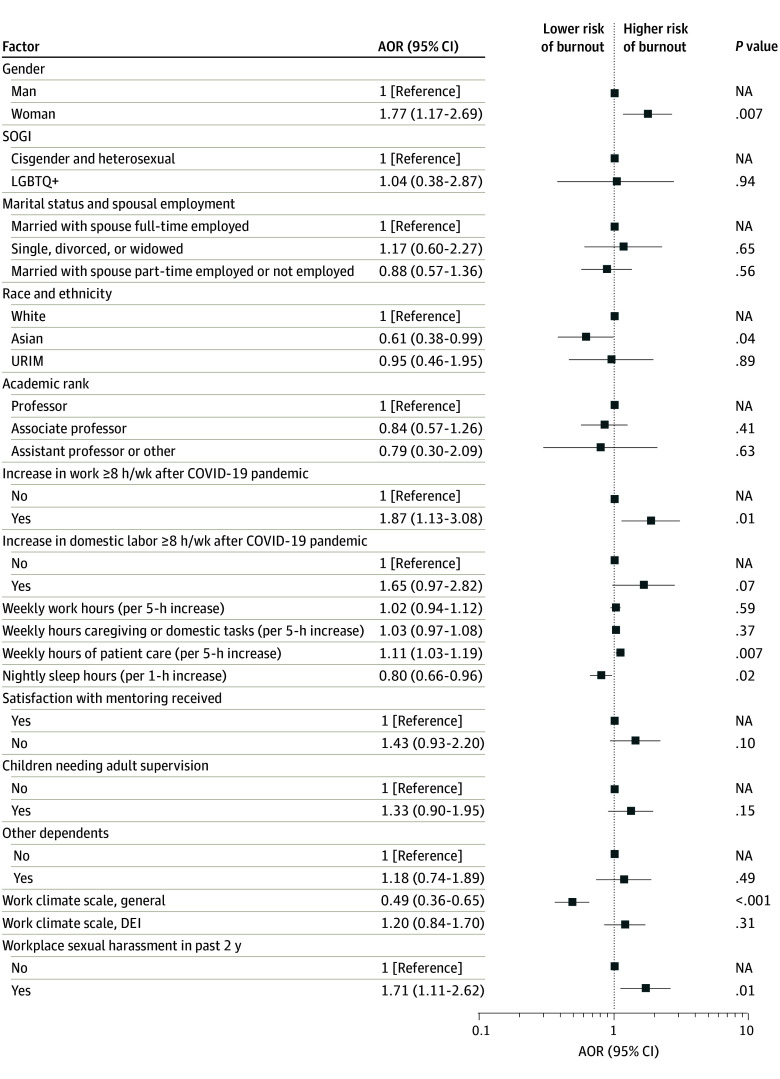
Multivariate Associations of Work-Related Burnout on the Copenhagen Burnout Inventory Races and ethnicities included in the underrepresented in medicine (URIM) category included American Indian or Alaska Native, Black or African American, Native Hawaiian or Other Pacific Islander, or multiple races other than Asian and White, which was categorized as Asian. AOR indicates adjusted odds ratio; DEI, diversity, equity, and inclusion; LGBTQ+, lesbian, gay, bisexual, transgender, or queer, with the plus holding space for the expanding definition of this term; NA, not applicable; and SOGI, sexual orientation and gender identity.

In a multivariable model of work-related burnout (Figure 3), burnout was again still significantly more likely for women than men (AOR, 1.77; [95% CI, 1.17-2.69]; *P* = .007), even after controlling for other covariates hypothesized to be potentially associated with the outcome. In addition to being more likely for women, work-related burnout was more likely among those reporting an increase of 8 or more work hours per week compared with pre–COVID-19 levels (AOR, 1.87 95% CI, 1.13-3.08]; *P* = .01), more weekly hours of patient care (AOR, 1.11 [95% CI, 1.03-1.19] for each 5-hour increase; *P* = .007), and having had an experience with sexual harassment in the workplace in the past 2 years (AOR 1.71 [95% CI, 1.11-2.62]; *P* = .01). Burnout was significantly less likely with more nightly sleep hours (AOR, 0.80 [95% CI, 0.66-0.96] for each 1-hour increase; *P* = .02) and with higher ratings on the general work climate scale (AOR, 0.49; [95% CI, 0.36-0.65] for each 1-point increase; *P* < .001).

As for sensitivity analyses, the multivariable models described herein report estimates from respondents with complete data for the burnout outcome and covariates. Although the percentage of data missing was low for any given covariate (<6% missing), when modeled together, missing data across covariates limited the model sample size to 690 of 841 respondents (82%) of the available sample. Estimates from models using multiple imputation reveal the same pattern of associations between the covariates modeled and burnout outcomes, with the associations similar in magnitude and statistical significance. The same pattern of meaningful associations was also estimated when the outcome was the continuous score (eAppendix in Supplement 1).

## Discussion

This survey study examined burnout among mid-career clinician-researchers working in academia using a validated instrument, the CBI, with an emphasis on gender differences. Indeed, we identified several gender-based differences; personal and work-related burnout were significantly more likely in women, even after controlling for several other factors that might both contribute to burnout and differ by gender. Factors associated with lower levels of both personal and work-related burnout in our multivariable model included more hours of nightly sleep and higher scores on the general work climate scale (ie, a more favorable work climate). Risk factors for work-related burnout included increased weekly hours of patient care and having an experience of sexual harassment at work in the past 2 years. Additionally, we assessed the impact of the COVID-19 pandemic with 2 variables due to its well-documented role on burnout among health care workers.^3,4^ In the multivariable analysis, we found that work-related burnout was associated with an increase of 8 or more work hours per week after the COVID-19 pandemic; these findings align with those of a recent study of over 40 000 health care workers that found that work overload due to COVID-19 was significantly associated with both burnout and intent to leave.^22^

A recent survey of K grant awardees from 2013 to 2019, including both physician investigators and doctoral-level scientists earlier in their careers than the sample that we studied, found that women were more likely than men to report high levels of burnout and were more likely to seriously consider leaving academic medicine.^23^ Those findings indicate that the patterns seen in the current study for mid-career clinician-researchers are consistent with those documented at earlier career stages.

A higher score on the general work climate scale was associated with lower risk of work-related burnout (OR, 0.49 [95% CI, 0.36-0.65] for each 1-point increase; *P* < .001). A favorable work climate on this scale is described as friendly, respectful, collegial, collaborative, cooperative, supportive, and welcoming. This association estimates a higher risk of burnout with unfavorable general work climate than that observed with a 5-hour increase in weekly work hours, in weekly caregiving or domestic task hours, or in weekly hours of patient care, all of which have been proposed as major potential contributors to burnout.^24,25^ Prior work from our group^9^ investigating this same cohort of physician-researchers found that women rated general climate and diversity climate more unfavorably than men, and were also more likely to experience gender harassment. These 3 aspects of culture were also found to adversely impact mental health in a multivariable analysis.^9^ The current findings complement and extend the prior work to demonstrate that experiences of climate serve as risk factors for burnout, suggesting an important target for interventions.

As pointed out by Powell et al^26^ in 2010, the need for workplace climate change is not a new idea in academic medicine; however, to our knowledge, this is the first study to show in a multivariable model an independent association with personal and work-related burnout for individuals practicing academic medicine. Wingard et al^27^ performed a single-institution 10-year longitudinal study from 2004 to 2015 of workplace climate–focused interventions, including routine data collection and dissemination, updates to policies and procedures focusing on equity and faculty support structures, and faculty development programming.^27^ Representation of faculty who were women and URIM increased over this time period, although salary equity between women and men was not achieved. In 2019, Burns et al^28^ examined how organizational culture affected both burnout and professional fulfillment at a single academic medical center. In that study, professional fulfillment and burnout were inversely correlated, and in particular in a multivariable linear regression model, reduced self-efficacy for addressing unprofessionalism was associated with burnout. Carapinha et al^29^ analyzed 2012 survey data spanning 13 medical schools and pointed out that work climate for women in academic medicine is institution-specific, finding that formal support structures for women, improved trust in leadership, and mitigation of discrimination and work-family conflict served to bolster a favorable climate for women.^29^ Moving forward, academic medical centers seeking to mitigate the consequences of burnout may seek to focus on improving work climate, with a specific focus on work climate for women.

### Limitations

The study limitations have been discussed in detail previously.^9^ In brief, although we had a relatively high response rate of 65%, a possibility of selection bias remains since invited individuals who chose to respond to the survey may have been more likely to have strong opinions about the studied topic. Nevertheless, the questionnaire included varied items and was not focused exclusively or even primarily on burnout. Similarly, individuals who had already left medicine were not included in the study, and their perspectives and experiences may have been even more extreme than those in our sample who continued on to more senior positions in academia. Additionally, this cohort might not be representative of mid-career academic physician-researchers who remained in academia but did not have the resources or mentorship that this cohort of former K grant awardees had in their early careers, limiting generalizability. Finally, because of the retrospective nature of some of the questions, such as those related to time allocation, recall bias may have impacted the results.

To perform the bivariate and multivariable analyses, we dichotomized the CBI personal and work-related burnout results with a cutoff value of 50. While this approach has been used previously with the CBI,^8,16,17,18^ other investigators have cautioned against using a cutoff because of the complex nature of burnout.^30^ We found it reassuring that sensitivity analyses did not suggest major differences in our findings based on the use of a cutoff vs a linear measure. Additionally, our sensitivity analyses found no major differences due to reporting estimates derived from the sample without missing covariates. Finally, it is worth noting that large variations in the way burnout is defined across the literature have resulted in difficulties in comparing burnout results across studies.^31^

## Conclusions

In this survey study of former K grant recipients now in mid-career, women were significantly more likely to experience burnout compared with men. In the multivariable analyses, gender remained a risk factor for both personal and work-related burnout, along with the general work climate. The COVID-19 pandemic, which has exacerbated preexisting differences in experiences by gender in academic medicine,^32^ has increased the need for evidence-based interventions to support women faculty throughout their careers and to monitor their experiences in the pandemic’s aftermath. Such interventions are essential to ensure access to all available talent and allow the field to reap the demonstrated benefits of workforce diversity.

## Notes

Supplement 1.
eAppendix

Supplement 2.
Data Sharing Statement
