# Supplementary material for: Burnout Among Mid-Career Academic Medical Faculty
Source: JAMA Netw Open. 2024 Jun 10;7(6):e2415593. doi: 10.1001/jamanetworkopen.2024.15593 (PMC11165383; doi:10.1001/jamanetworkopen.2024.15593)

Supplemental Online Content

Paradis KC, Kerr EA, Griffith KA, et al. Burnout among mid-career academic medical faculty. *JAMA Netw Open*. 2024;7(6):e2415593. doi:10.1001/jamanetworkopen.2024.15593

eAppendix

This supplemental material has been provided by the authors to give readers additional information about their work.

eAppendix

Sensitivity Analyses #1

The original dichotomous 'burned-out' models using a Copenhagen Burnout Inventory (CBI) burnout score cutoff of ≥50 were estimated using logistic regression and a case-wise deletion sample population. This method estimates the associations between explanatory covariates and the 'burned-out' outcome using data for only those respondents with complete data (i.e., without item non-response). This framework omits any respondent's data if the outcome or explanatory covariates were missing. Although item non-response for the survey was low (<6%) for any particular explanatory covariate as shown in Table 1, when assessed jointly in a multivariable model, the sample size on which the models were estimated was reduced to 690/841 complete cases, which is 82.0% of the sample possible. If the reason for item non-response is related to the outcome, in this case to burnout, then analysis of complete-case data may cause bias and misleading results. In order to ascertain whether or not that could be the situation in this analysis, a sensitivity analysis was constructed by imputing any missing data in the model covariates using fully conditionally specified (FCS) chained equations. This method uses each subject's available (non-missing) data to predict missing data using statistical models. This method is duplicated in practice to create 5 imputed datasets, in order to understand the statistical uncertainty this imputation prediction introduces. The final associations are the average model estimates from these 5 imputed datasets. FCS chained equation multiple imputation is a statistically accepted method to best estimate associations when missing data exists (White, I. R., P. Royston, and A. M. Wood. 2011. Multiple imputation using chained equations: Issues and guidance or practice. Statistics in Medicine 30: 377–399; https://doi.org/10.1002/sim.4067). FCS chained equation multiple imputation was calculated using STATA version 16.1 (StataCorp, College Station, TX).

The multiply imputed analyses were extremely similar to the complete case analyses reported and suggest that item non-response (missing data) did not bias the estimated associations reported. In particular both the multiply imputed analysis and complete cases analysis for personal burnout estimated that gender, nightly sleep hours, and the general work climate scale were significantly associated with similar magnitudes of associations (See Figures 3 and S1). In addition, the complete case analysis estimated weekly hours of patient care was significantly associated with personal burnout (odds ratio (OR) 1.07, 95% confidence interval (CI) 1.001-1.15, for each 5 hour increase, p=0.044). However, the multiply imputed analysis estimated a similar OR that was not strictly significant (OR 1.05, 95%CI 0.99-1.13, for each 5 hour increase, p=0.118). Similar to personal burnout, multiply imputed analysis and complete cases analysis for work-related burnout were extremely similar, and both estimated gender, disruption of work hours, weekly patient care hours, nightly sleep hours, the general work climate scale, and sexual harassment experience were significantly associated and to a similar extent (See Figures 4 and S2). In addition, the multiply imputed model estimated that disruption of domestic labor hours significantly associated with work-related burnout (OR 1.66, 95%CI 1.001-2.73, p=0.048). However, the complete case analysis estimated a similar OR that was not strictly significant (OR 1.65, 95%CI 0.96-2.82, p=0.066).

Associations from a multivariable model using multiple imputation of personal burnout by the Copenhagen Burnout Inventory (CBI). Sample includes 841 mid-career academic medical faculty who received K-Awards between 2006-2009 and responded to a 2021-2022 survey. CL, confidence limit; LCL, lower confidence limit; UCL, upper confidence limit; SOGI, sexual orientation and gender identity; LGBTQIA+, lesbian, gay, bisexual, transgender, queer, intersex, asexual, with the plus holding space for the expanding definition of this term.

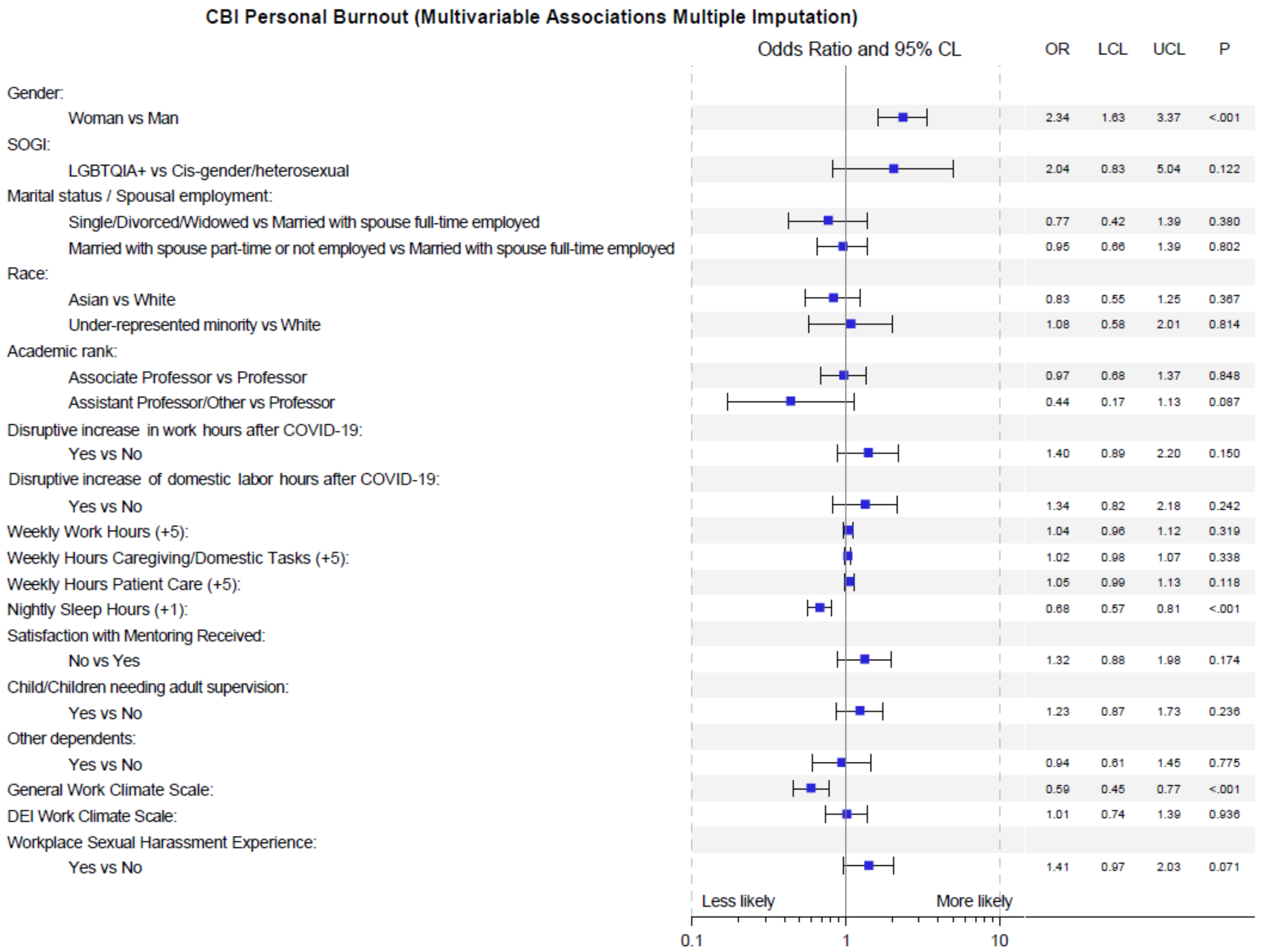

Associations from a multivariable model using multiple imputation of work-related burnout by the Copenhagen Burnout Inventory (CBI). Sample includes 841 mid-career academic medical faculty who received K-Awards between 2006-2009 and responded to a 2021-2022 survey. CL, confidence limit; LCL, lower confidence limit; UCL, upper confidence limit; SOGI, sexual orientation and gender identity; LGBTQIA+, lesbian, gay, bisexual, transgender, queer, intersex, asexual, with the plus holding space for the expanding definition of this term.

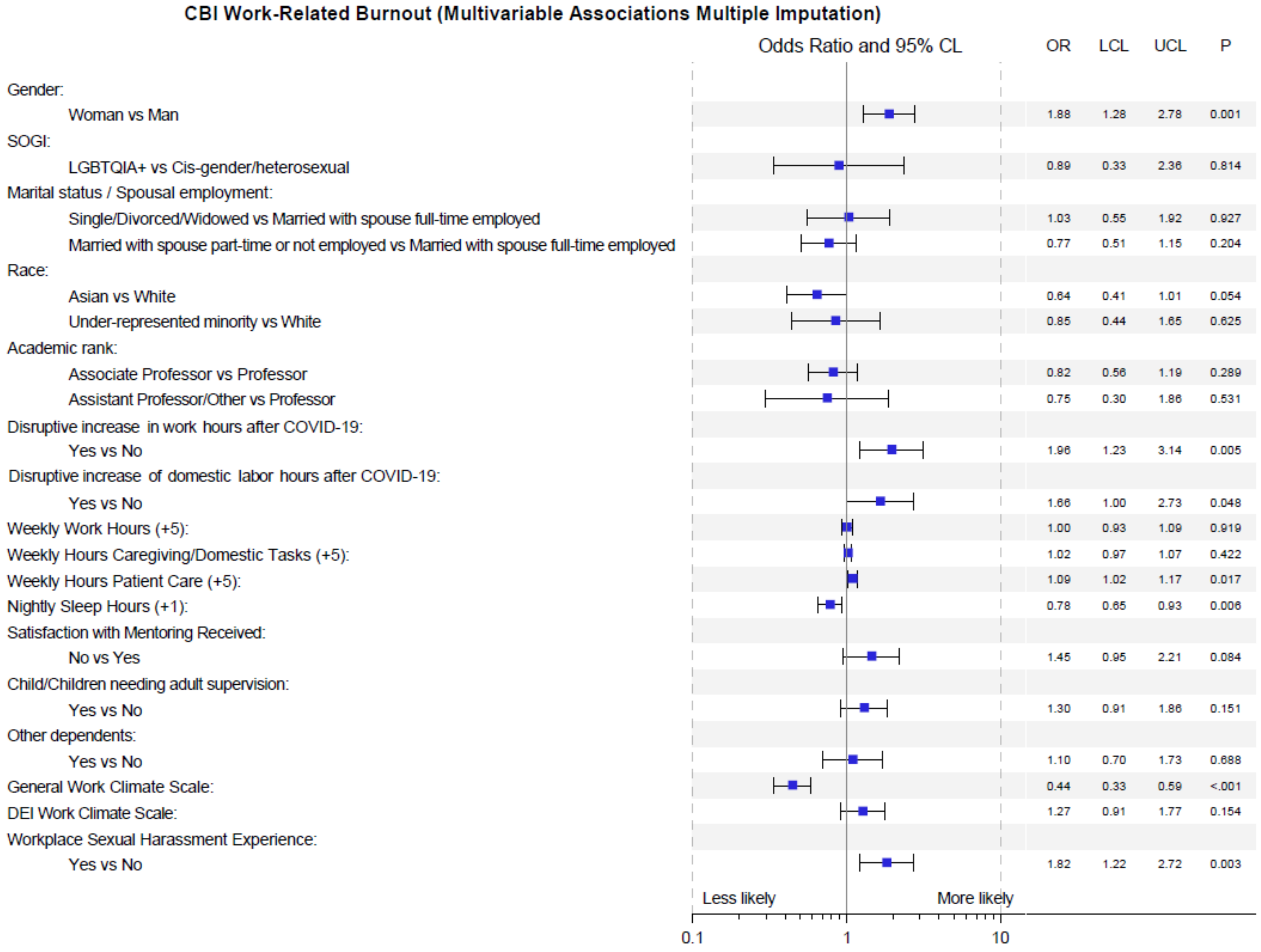

Sensitivity Analysis #2

Rather than dichotomizing the outcome into 'burned-out' or not, one can estimate the associations between continuous CBI score (0-100) for both personal and work-related burnout, to covariates using linear regression. Therefore, a second sensitivity analysis was conducted using continuous CBI score to the dichotomous models primarily presented. Like the first sensitivity analysis, these linear regression models can be estimated upon the FCS chained equation multiple imputation data using the continuous scores as the outcomes of interest. The pattern of significant effects can be compared between the multiply imputed results for the dichotomous outcome to assess the impact of choosing a cut-point for significant effect. The continuous covariates were centered at their median values so that the intercept from the continuous linear is interpretable. The interpretation of the intercept is the average score for a respondent that has median values for the continuous covariates and the reference values for categorical covariates. Specifically, the intercept value would be the estimated CBI score for a man, who was cis-gender/heterosexual, white, rank of professor, did not have disruptive increase (of 8 or more hours) of work hours as compared to before the COVID outbreak, did not have disruptive increase (of 8 or more hours) of domestic labor hours as compared to before the COVID outbreak, who worked 60 weekly work hours, who had 21 hours of weekly caregiving/domestic labor, who had 12 weekly hours of patient care, who gets 7 hours of nightly sleep, who is married/domestic partnered with a full-time employed spouse, was satisfied with mentoring received, did not have a child or children requiring adult supervision, did not have other dependents, had a general work climate score of 4, had a DEI work climate score of 4, and did not experience sexual harassment.

The multivariable linear regression multiply imputed model for personal burnout found gender, nightly sleep hours, child/children needing adult supervision, the general work climate scale, and sexual harassment experience were significantly associated with the continuous CBI burnout score. This is similar to the finding from the dichotomous cut-point model with the exception of child/children needing adult supervision and sexual harassment, which each were estimated to positively associate to burnout, but not to a strictly significant level ($p<0.05$).

The multivariable linear regression multiply imputed model and the multivariable dichotomous logistic regression multiply imputed model for work-related burnout both identified exactly the same covariates as being significantly associated: gender, disruptive increase of work hours, disruptive increase of domestic labor hours, weekly patient care hours, nightly sleep hours, general work climate score, and sexual harassment experience.

**Linear regression multiple variable model using multiply imputed data for CBI personal burnout score**			
**Covariate**	**Estimate**	**95%CI**	**P-value**
Intercept: (Continuous)	30.52	27.39, 33.65	<0.0001
Gender:	.		.
Man	0.00		.
Woman	7.12	4.45, 9.78	<0.0001
LGBTQIA+:	.		.
LGBTQIA+	3.56	-3.15, 10.27	0.30
Cis-gender/heterosexual	0.00		.
Race:	.		.
White	0.00		.
Asian	0.14	-2.81, 3.09	0.93
Under-represented minority	0.90	-3.72, 5.51	0.70
Academic rank:	.		.
Professor	0.00		.
Associate professor	0.75	-1.81, 3.30	0.57
Assistant professor	-2.05	-8.16, 4.06	0.51
Disruptive increase of work hours after COVID:	.		.

Linear regression multiple variable model using multiply imputed data for CBI personal burnout score			
**Covariate**	**Estimate**	**95%CI**	**P-value**
No	0.00		.
Yes	2.64	-0.84, 6.12	0.14
Disruptive increase of domestic labor hours after COVID:	.		.
No	0.00		.
Yes	2.03	-1.61, 5.66	0.27
Weekly work hours (Continuous, centered at 60 hours):	.		.
+5:	0.26	-0.31, 0.82	0.37
Weekly hours caregiving/domestic labor (Continuous, centered at 21 hours):	.		.
+5:	0.16	-0.20, 0.51	0.39
Weekly hours patient care (Continuous, centered at 12 hours):	.		.
+5:	0.33	-0.16, 0.83	0.18
Nightly sleep hours (Continuous, centered at 7 hours):	.		.
+1:	-3.02	-4.27, -1.78	<0.0001
Marital status/spousal employment:	.		.
Single/Divorced/Widowed	0.79	-3.69, 5.26	0.73
Married/Not or part-time employed spouse	1.56	-1.13, 4.25	0.26
Married/Full-time employed spouse	0.00		.
Satisfaction with mentoring received:	.		.
No	1.25	-1.74, 4.24	0.41
Yes	0.00		.
Child/Children needing adult supervision:	.		.
No	0.00		.
Yes	3.31	0.82, 5.79	0.009
Other dependent(s):	.		.
No	0.00		.
Yes	0.45	-2.70, 3.59	0.78
General work climate scale (Continuous, centered at 4):	.		.
+1	-4.94	-6.95, -2.93	<0.0001
DEI work climate scale (Continuous, centered at 4):	.		.
+1	0.61	-1.77, 2.99	0.62
Sexual harassment experience:	.		.
No	0.00		.
Yes	3.97	1.27, 6.66	0.004

Linear regression multiple variable model using multiply imputed data for CBI work-related burnout score			
**Covariate**	**Estimate**	**95%CI**	**P-value**
Intercept: (Continuous)	28.04	25.02, 31.05	<0.0001
Gender:	.		.
Man	0.00		.
Woman	5.69	3.07, 8.31	<0.0001
LGBTQIA+:	.		.
LGBTQIA+	1.50	-4.93, 7.92	0.65
Cis-gender/heterosexual	0.00		.
Race:	.		.
White	0.00		.
Asian	-3.15	-6.07, -0.22	0.03
Under-represented minority	-1.62	-6.14, 2.90	0.48
Academic rank:	.		.
Professor	0.00		.
Associate professor	0.14	-2.36, 2.64	0.91
Assistant professor	-1.16	-7.14, 4.82	0.70
Disruptive increase (8 or more) of work hours after COVID:	.		.
No	0.00		.
Yes	5.97	2.59, 9.34	0.0005
Disruptive increase (8 or more) of domestic labor hours after COVID:	.		.
No	0.00		.
Yes	3.56	-0.01, 7.13	0.05
Weekly work hours (Continuous, centered at 60 hours):	.		.
+5:	0.19	-0.35, 0.73	0.50
Weekly hours caregiving/domestic labor (Continuous, centered at 21 hours):	.		.
+5:	0.11	-0.24, 0.46	0.54
Weekly hours patient care (Continuous, centered at 12 hours):	.		.
+5:	0.64	0.17, 1.11	0.008
Nightly sleep hours (Continuous, centered at 7 hours):	.		.
+1:	-1.63	-2.86, -0.41	0.009
Marital status/spousal employment:	.		.
Single/Divorced/Widowed	1.59	-2.86, 6.04	0.48
Married/Not or part-time employed spouse	1.37	-1.27, 4.01	0.31
Married/Full-time employed spouse	0.00		.

Linear regression multiple variable model using multiply imputed data for CBI work-related burnout score			
**Covariate**	**Estimate**	**95%CI**	**P-value**
Satisfaction with mentoring received:	.		.
No	2.14	-0.74, 5.01	0.14
Yes	0.00		.
Child/Children needing adult supervision:	.		.
No	0.00		.
Yes	1.58	-0.82, 3.99	0.20
Other dependent(s):	.		.
No	0.00		.
Yes	-0.51	-3.60, 2.59	0.75
General work climate scale (Continuous, centered at 4):	.		.
+1	-7.35	-9.30, -5.39	<0.0001
DEI work climate scale (Continuous, centered at 4):	.		.
+1	0.37	-1.93, 2.67	0.75
Sexual harassment experience:	.		.
No	0.00		.
Yes	4.77	2.18, 7.36	0.0003

CBI Personal Burnout Bivariate Analysis Summary, Adjusted for Multiple Testing Via Method of Holm

In examining bivariate associations of personal CBI score, burnout was significantly more likely for women than men (OR 2.35, 95%CI 1.72-3.22, p<0.001), in the presence of a disruptive increase in work hours after COVID-19 (OR 2.11, 95%CI 1.43-3.10, p=.002), in the presence of a disruptive increase of domestic labor hours after COVID-19 (OR 2.18, 95%CI 1.45-3.27, p=.002), with higher weekly work hours (OR 1.13, 95%CI 1.06-1.20, with each 5-hour increase, p=0.001), with higher weekly caregiving/domestic tasks hours (OR 1.07, 95%CI 1.03-1.11, with each 5-hour increase, p=0.009), when not being satisfied with mentoring received (OR 2.00, 95%CI 1.42-2.80, p<0.001), and when having experienced sexual harassment (OR 2.20, 95%CI 1.59-3.04, p<0.001). Burnout was significantly less likely with higher nightly sleep hours (OR 0.65, 95%CI 0.55-0.76, with each 1-hour increase, p<0.001), less likely with an improved general work climate score (OR 0.52, 95%CI 0.43-0.62, with each 1-point increase, p<0.001), and less likely with an improved DEI work climate score (OR 0.52, 95%CI 0.42-0.64, with each 1-point increase, p<0.001).

Associations from a series of bivariate models of personal burnout by the Copenhagen Burnout Inventory (CBI). Sample includes 841 mid-career academic medical faculty who received K-Awards between 2006-2009 and responded to a 2021-2022 survey. CL, confidence limit; LCL, lower confidence limit; UCL, upper confidence limit; SOGI, sexual orientation and gender identity; LGBTQIA+, lesbian, gay, bisexual, transgender, queer, intersex, asexual, with the plus holding space for the expanding definition of this term. Standard p-values and adjusted p-values for multiple testing using the method of Holm are reported.

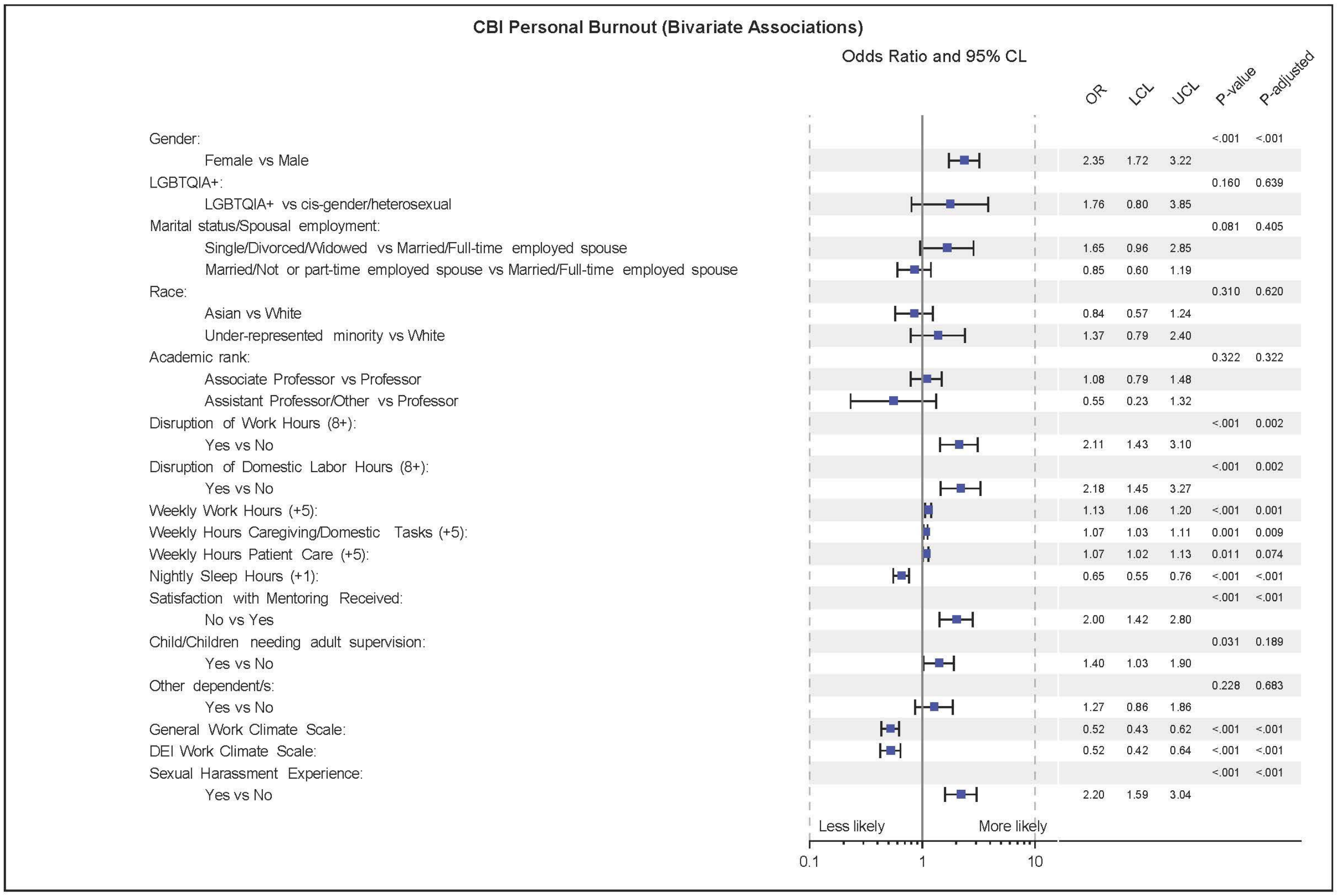

CBI Work-related Burnout Bivariate Analysis Summary, Adjusted for Multiple Testing Via Method of Holm

In examining bivariate associations of work-related CBI score, burnout was significantly more likely for women than men (OR 2.05, 95%CI 1.48-2.84, p<0.001), in the presence of a disruptive increase in work hours after COVID-19 (OR 2.72, 95%CI 1.84-4.02, p<0.001), in the presence of a disruptive increase of domestic labor hours after COVID-19 (OR 2.41, 95%CI 1.60-3.64, p<0.001), with higher weekly work hours (OR 1.15, 95%CI 1.08-1.23, with each 5-hour increase, p<0.001), with higher weekly caregiving/domestic tasks hours (OR 1.08, 95%CI 1.03-1.12, with each 5-hour increase, p=0.003), with higher weekly patient care hours (OR 1.10, 95%CI 1.04-1.17, with each 5-hour increase, p=0.004), when not being satisfied with mentoring received (OR 2.28, 95%CI 1.61-3.23, p<0.001), and when having experienced sexual harassment (OR 2.84, 95%CI 1.99-4.05, p<0.001). Burnout was significantly less likely with higher nightly sleep hours (OR 0.72, 95%CI 0.62-0.85, with each 1-hour increase, p<0.001), less likely with an improved general work climate score (OR 0.44, 95%CI 0.36-0.53, with each 1-point increase, p<0.001), and less likely with an improved DEI work climate score (OR 0.52, 95%CI 0.42-0.64, with each 1-point increase, p<0.001).

Associations from a series of bivariate models of work-related burnout by the Copenhagen Burnout Inventory (CBI). Sample includes 841 mid-career academic medical faculty who received K-Awards between 2006-2009 and responded to a 2021-2022 survey. CL, confidence limit; LCL, lower confidence limit; UCL, upper confidence limit; SOGI, sexual orientation and gender identity; LGBTQIA+, lesbian, gay, bisexual, transgender, queer, intersex, asexual, with the plus holding space for the expanding definition of this term. Standard p-values and adjusted p-values for multiple testing using the method of Holm are reported.

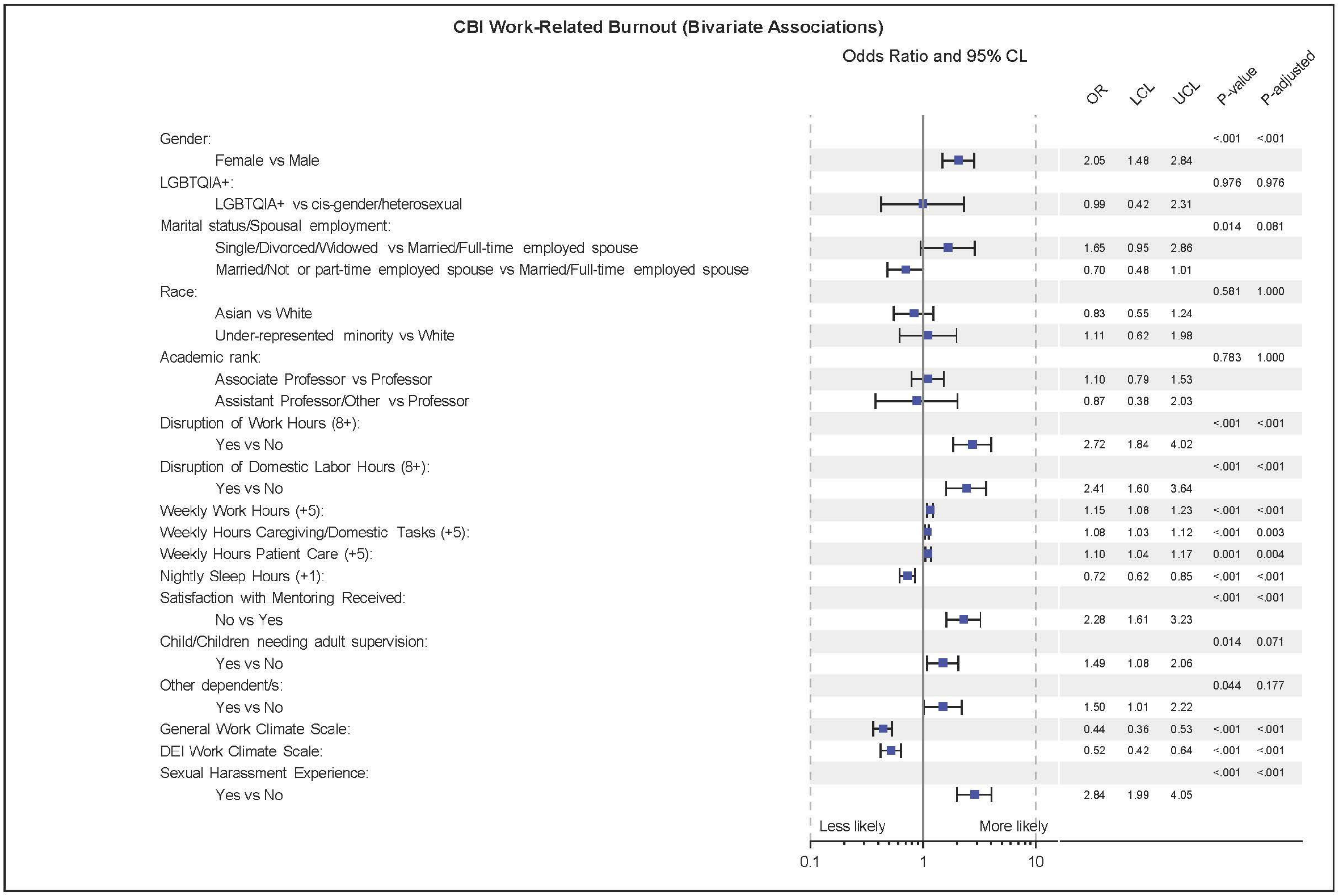

Supplement: Supplement 1. — eAppendix [file jamanetwopen-e2415593-s001.pdf]
