# Supplementary material for: Burnout Among Mid-Career Academic Medical Faculty
Source: JAMA Netw Open. 2024 Jun 10;7(6):e2415593. doi: 10.1001/jamanetworkopen.2024.15593 (PMC11165383; doi:10.1001/jamanetworkopen.2024.15593)
Supplement: Supplement 2. — Data Sharing Statement [file jamanetwopen-e2415593-s002.pdf]

Data Sharing Statement

Paradis. Burnout Among Mid-Career Academic Medical Faculty. *JAMA Netw Open*. Published June 10, 2024. doi:10.1001/jamanetworkopen.2024.15593

Data
Data available: No

Additional Information
Explanation for why data not available: Data in this study will not be shared to protect participant confidentiality.
